# The effects of Transcutaneous Electrical Nerve Stimulation on postural control in patients with chronic low back pain

**Published:** 2015

**Authors:** Z Rojhani-Shirazi, T Rezaeian

**Affiliations:** * Physical Therapy, School of Rehabilitation Sciences, Shiraz University of Medical Sciences, Shiraz, Iran; ** Student Research Committee, School of Rehabilitation Sciences, Shiraz University of Medical Sciences, Shiraz, Iran

**Keywords:** chronic low back pain, postural control, Transcutaneous Electrical Nerve Stimulation

## Abstract

**Objective:** The effects of transcutaneous electrical nerve stimulation (TENS) on postural control in patients with low back pain which is not well known. This study aimed to evaluate the effects of TENS on postural control in chronic low back pain.

**Methods: **This study was an experimental research design. Twenty-eight patients with chronic LBP (25-45 Y/ O) participated and by using a random allocation, were divided to samples who participated in this study. The mean center of pressure (COP) velocity and displacement were measured before, immediately and 30 min after the intervention. The tests were done with eyes open and closed on a force platform. Sensory electrical stimulation was applied through the TENS device. The descriptive statistics, independent sample T-test and ANOVA with repeated measurement on time were used for data analysis.

**Results: **The results of the present study demonstrated that the application of the sensory electrical stimulation in chronic LBP patients showed a statistically significant improvement in postural control in Medio-lateral direction with no corresponding effect on the anterior-posterior direction immediately following the TENS application and 30 minutes after it in closed eyes conditions as compared to baseline. The application of TENS decreased the displacement and velocity of COP (p≤0.05), 30 minutes after the application of sensory electrical stimulation. The results showed that the mean displacement and velocity of COP decreased in eyes open position (p≤0.05). Also, immediately and 30 minutes after the application of sensory electrical stimulation, COP displacement and velocity in ML direction with eyes closed significantly decreased in the intervention group in comparison with control group (p≤0.05).

**Conclusion: **The application of TENS in patients with chronic low back pain could improve postural control in these patients.

## Introduction

Low back pain (LBP) is a very common disorder, and studies have shown that more than 80% of the people will suffer from LBP over their lifetime [**1**-**3**]. Many of the acute LBP patients present this disorder during 4 weeks, but recurrence of pain episodes is common [**4**]. 10–40% of the individuals with LBP develop into having chronic LBP and are considered as the most costly musculoskeletal disorder for society [**1**,**4**].

One of the most important factors in the genesisand persistence of nonspecific LBP isstability and control of the spine.Studies in LBP patients have indicated impairments in the deep trunk muscles (e.g. transverses abdominis and multifidus) responsiblefor maintaining the stabilityof the spine [**5**,**6**].

The human postural system is controlled by the coordination of the three sensory sources including visual, vestibular and proprioceptive inputs. These systems provide information about the status and movements of the body in thespace and continuously transmit and generate enough force for controlling and maintaining balance in various situations [**7**,**8**]. Therefore,it is clear that a disruption in any of these sensory systems will affect the postural control.The previous studies revealed that the postural control in subjects with chronic low back pain and some components of these systems such as the physiology of afferent and efferent nerves may be affected [**9**,**10**]. This damage can lead to a significantly greater sway in the upright standing, which may play a role in the recurrence of low back pain[**9**].

A poor postural control mechanism in subjects with low back pain is not known yet completely [**11**]. Proprioceptive inputs or sensory integration deficits have been suspected as the possible causes of balance impairments in people with chronic low back pain although there is no sufficient evidence in this issue [**12**]

Since subjects with low back pain exhibit postural control impairment, researchers could find a new insight into rehabilitation for postural control impairment in these patients.

One potential means of further enhancing the improvement in proprioception is subsensory stochastic resonance (SR) electrical stimulation. SR stimulation is a type of electrical or mechanical stimulation with an alternating electric field that, at a subsensory level, has been shown to enhance the detection and transmission of weak sensory signals [**14**,**15**].Stochastic resonance is thought to alter the transmembrane potential of neurons, causing the cell to depolarize and make it more likely that an action potential will result [**13**]. It has shown promise in improving balance in various populations including the elderly [**17**,**20**]those with diabetic neuropathy [**25**], and those recovering from stroke [**28**]. As somatosensory feedback is an important component to the balance control system, it has been theorized that the improved balance observed with SR stimulation is a result of the enhanced proprioceptive input [**20**].

In 2002, Gravelle et al. tested the effect of SR with low-level electrical noise, applied at the knee, on balance control in healthy elderly volunteers. They showed that low-level input noise (electrical or mechanical) could enhance the sensitivity of the human somatosensory system. The results suggested that the imperceptible electrical noise, when applied to the knee, could enhance the balance performance of healthy older adults [**19**,**20**].

In 2002, Dhruv and colleagues showed that low-level electrical noise could significantly improve fine-touch sensitivity on the plantar surface of the foot in the elderly by using Semmes-Weinstein monofilaments. Therefore, the study suggested that the electrical noise-based techniques might enable people to overcome functional difficulties due to age-related sensory loss [**16**,**17**].

Transcutaneous electrical nerve stimulation (TENS) is one of these modalities that can improve neuromuscular function/pain status and therefore its benefits in low back pain patients, who experience pain and muscle weakness around the pelvis, trunk and lower limbs leading to low back pain.

TENS, which involves the pulsatile stimulation of sensory fibers, is used primarily for the purpose of pain modulation in physiotherapy [**21**]. Different types of TENS treatment are often referred to as Hi-TENS and Low-TENS. TENS for pain control usually applies high frequency stimulation; while for excitatory effects of sensory inputs on the motor system, lower frequencies (10 Hz), have generally been used. Studies using Trans-Cranial Magnetic Stimulation (TMS) have obtained evidence that the application of TENS at different body sites influences cortico-motor excitability. Therefore, the application of TENS may interfere in the modulation of cortical motor responses including postural control responses [**22**]. Fraser et al. stated that the motor effects are likely to critically depend on the frequency of sensory stimulation [**23**].

In 2002, Gravelle et al.investigated the effect electrical noise, used at the knee, on postural control in healthy older adults. They showed that electrical or mechanical noise could improve the human somatosensory system. The results showed that when used to the knee, the electrical noise could improve the postural performance of healthy elderly people [**20**].

The application of TENS to the neck muscles in patients with hemispatial neglect has been shown to improve spatial orientation and postural control [**24**].

Therefore, TENS is usually used with sensory threshold or supra-threshold amplitude compared with sub-threshold sensorimotor signals of SR. TENS seems to be more acceptable than SR stimulation among patients because of its perceptible stimulation current but its effect on postural control is unknown [**25**].

Several investigations on the effects of SR have reported improvements in balance control when an electrical or mechanical noise was applied [**20**,**28**,**29**,**44**].

To the best of our knowledge, no study has investigated the possible effect of TENS onpostural control in patients with low back pain. Therefore, the purpose of thisstudy was to evaluate the acute effect of application of TENS on postural control in chronic low back patients.

## Materials and methods

**Participants**

Twenty-eight chronic low back pain (CLBP) patients (24 women, 4 men) participated in this study and were matched for age, weight, height, and BMI. All the participants were included in this study if they had a diagnosis of chronic low back pain from a physician who recruited them from referrals of local physiotherapy clinics in Shiraz. Low back pain is defined as pain in the area between the 12th rib and the gluteal folds. All the patients had mild to moderate (0 to 40%) disability in Oswestry questionnaire and their age range was between 25-45 years.

The inclusion criteria were age between 25-45 years, localized back pain, lasting for more than 6 months and radiating no further than the buttock, no previous history of sciatica or other radicular involvement, at least 3 of the 10 visual analog scales (VAS) and mild to moderate (0 to 40%) disability in Oswestry questionnaire

The exclusion criteria were the history of neurological signs such as sensory or motor deficits, likeparalysis or vestibular system impairment, dizziness and medication with known effects onbalance, history of spinal surgery, rheumatic diseases, diabetes, mental disorders, pregnancy, lower extremity injuries, and neuromuscular diseases.

**Procedures**

After a visit and check of the inclusion criteria, all the participants signed an informed consent form approved by the ethics committee of Shiraz University of Medical Sciences and then participated in this study.

Through non-random sampling, twenty-eight CLBP patients (25-45 y/o) participated in this study. Then, by using block randomization, they were allocated in the intervention and control groups. Fourteen subjects received TENS, while the remaining fourteen received sham intervention. In addition, the patients did the tests with eyes open and closed. A randomized block design was used to determine the test order. In the intervention group, measurements were performed with eyes open and closed on a force platform, before, immediately, and 30 minutes after the intervention. It should be mentioned that all measurement tests were repeated twice. In the control group, the tests were done similar to the intervention group, but they received sham electrical stimulation.

**Postural Control**

To measure the postural control, a force platform (Kistler Instrument®, Switzerland), sampling at 100 Hz was used. The anteroposterior (AP) and mediolateral (ML) displacements (mm) of COP were stored for analysis. Raw data were exported to Visual 3D® software and filtered by using a fourth order low-pass Butterworth filter with a cut off frequency of 12 Hz.

Participants stood barefoot in a double leg stance with eyes open and closed on the force plate for two trials of 20s. Participants were asked to stand relaxed, immobile.During the double leg stance condition, they were instructed to stand comfortable with normal posture and their feet approximately at the pelvis width and the arms were hanging loosely by their sides [**26**]. They were standing in an upright position with eyes open, focusing on a target placed at the eye level, two meters in front of them. Postural stability measurements were recorded before, immediately, and 30minutes after the intervention.

**Intervention Group**

**Fig. 1:**
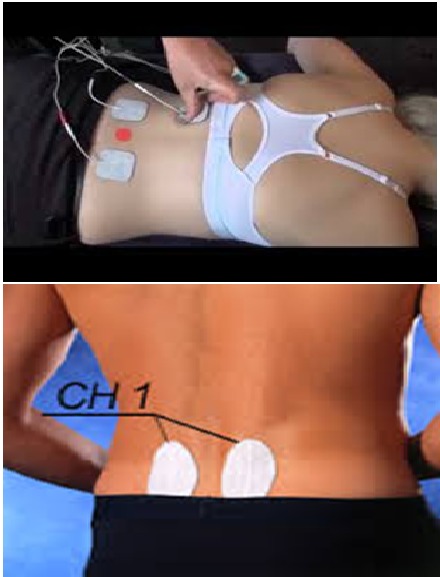
Photos for electrode placement and intervention protocol

In order to apply the TENS technique, the subjects were positioned prone on a treatment bench; then, electrical stimulation was applied via an electrical stimulator device (low frequency TENS with a duration of 250 µs and 7 HZ frequency) through pairs of electrodes placed 1cm away from the spinus process L1 and L5 in each sides for 15 minutes at tolerance level. The data were re-evaluated before, immediately, and 30minutes after the intervention (**Fig. 1**).

**Control Group**

The control group received sham electrical stimulation. The subjects were positioned prone on a treatment bench; then, electrical stimulation was applied via an electrical stimulator device (low frequency TENS with a duration of 250 µs and 7 HZ frequency) through pairs of electrodes placed 1cm away from the spinus process L1 and L5 in each sides for 15 minutes but intensity was zero. The data were re-evaluated before, immediately, and 30minutes after the intervention (**Fig. 1**).

**Statistical analysis**

The data were analyzed by using SPSS, version 16. The homogeneity of the variance of COPvariables was assessed by Shapiro-Wilk test of normality. The descriptive statistics, independent sample T-test, repeated measurement, and ANOVA with repeated measurement on time were used for data analysis. Post-hoc test was also applied wherever necessary. The level of significance for all the tests was set at 0.05.

## Results

**Displacement and velocity of cop**

All the participants were able to stand for 20s during the test. There were no significant differences between the two groups regarding the anthropometric data. The mean age, BMI, pain score and disability score of these patients were 30.89 (+/-7.29), 27.05 years (+/-2.74), 4.50(+/-1.17) and 20.91(+/-11.07) respectively Error! Reference source not found.. The patients presenting with Oswestry Questionnaire ranging from 0 to 40% were considered to be mild to moderately disable, and were not excessively obese or elderly.(**Table 1**)

**Table 1:** Mean (+/-SD) demographic information for all test subjects

Variable	TENS(n = 14)	Place boTENS (n = 14)	Total
Age	31.64 +/- 12.49	30.57 +/-7.51	30.89 +/- 7.29
Height (cm.)	164 +/- 0.05	160 +/- 0.05	162 +/- 0.05
Weight (kg.)	66.85 +/-12.06	57.85 +/- 12.29	62.35 +/- 1.27
BMI (kg/ m2)	27.69 +/- 2.97	26.40 +/- 2.42	27.05 +/- 2.74
Gender (Female/ Male)	12.2	12.2	24.4
VAS	4.64 +/- 1.08	4.35 +/- 1.27	4.50 +/- 1.17

**Electrical stimulation and postural control in low back pain patients**

ANOVA was used to examine the effect of time (pre, post and follow-up) on each group separately, and Independent T-test to compare between groups (at each assessment,and at each condition i.e. eyes open and closed). However, the correct model for this design would be a 2 (groups) × 2 (conditions) × 3 time (pre, post, follow-up) ANOVA. We chose to do 2×3 for the eyes open and the eyes closed separately. Also, we used a repeated measurement to evaluate the effect of time (pre, post and follow-up) between group differences over time.

The repeated measure ANOVA for within group differences and Bonferroni Post Hoc was used. The result showed that immediately and 30 minutes after the application of sensory electrical stimulation, COP displacement and velocity in ML direction with eyes closed (p=0.001) took place (**Table 2**).

According to Post Hoc, it was reported that immediately after the application of sensory electrical stimulation, COP displacement (before: 2.95 +/- 0.83 mm, immediately: 2.27+/-0.45 mm) (p= 0.01) and COP velocity in ML direction with eyes closed (before: 0.092 +/- 0.02mm/s, immediately: 0.072 +/- 0.01mm/s) (p= 0.02) significantly decreased in the intervention group as compared to the baseline.

Also, after 30 minutes, the displacement of COP in ML direction with eyes closed (before: 2.95 +/- 0.83 mm, 30min: 2.21+/- 0.52mm) (p= 0.007) and velocity of COP in ML direction with eyes closed (before: 0.092 +/- 0.02mm/s, 30minutes: 0.068+/- 0.01) (p= 0.004) significantly decreased in the intervention group as compared to the baseline.

The results of the independent T-test for between group differences showed that immediately after the application of sensory electrical stimulation, COP displacement, and velocity in ML direction with eyes closed significantly decreased in the intervention group in comparison with the control group (**Table 2**).

After 30 minutes, COP displacement and velocity in ML direction with eyes closed significantly decreased in the intervention group compared to the control group (**Table 2**).

**Table 2:** The comparison of the mean COP displacement and velocity before, immediately, and 30 minutes after the application of TENS with eyes closed and open

variables			TENS	Placebo-TENS	P- value**
COP displacement (mm) Eye Open	ML	Before	2.92 ± 1.07	3.56 ± 1.97	0.30
		Immediate	2.40 ± 0.78	3.42 ± 1.66	0.05
		30 After	2.32 ± 0.79	3.37 ± 2.14	0.09
		*P- value	0.08	0.85	
	AP	Before	2.19 ± 0.94	2.31 ± 1.27	0.78
		Immediate	2.18 ± 1.15	1.98 ± 0.79	0.59
		30 After	1.85 ± 1.73	2.89 ± 2.27	0.18
		*P- value	0.55	0.25	
COP displacement (mm) Eye Closed	ML	Before	2.95 ± 0.83	3.57 ± 1.63	0.22
		Immediate	2.27 ± 0.45	3.54 ± 1.91	0.02#
		30 After	2.21 ± 0.52	3.50 ± 2.21	0.04#
		*P- value	0.001#	0.97	
	AP	Before	1.98 ± 1.2	2.02 ± 1.6	0.93
		Immediate	1.81 ± 0.72	2.18 ± 1.43	0.39
		30 After	1.66 ± 0.58	2.52 ± 0.67	0.22
		*P- value	0.34	0.25	
COP Velocity (mm/ s) Eye Open	ML	Before	0.09 ± 0.03	0.11 ± 0.06	0.24
		Immediate	0.072 ± 0.02	0.10 ± 0.05	0.05
		30 After	0.072 ± 0.02	0.10 ± 0.07	0.10
		*P- value	0.14	0.82	
	AP	Before	0.06 ± 0.03	0.071 ± 0.04	0.80
		Immediate	0.06 ± 0.03	0.06 ± 0.02	0.57
		30 After	0.058 ± 0.05	0.072 ± 0.04	0.48
		*P- value	0.65	0.34	
COP Velocity (mm/ s) Eye Closed	ML	Before	0.09 ± 0.02	0.11 ± 0.05	0.10
		Immediate	0.072 ± 0.01	0.11 ± 0.06	0.03#
		30 After	0.068 ± 0.01	0.11 ± 0.07	0.04#
		*P- value	0.001#	0.78	
	AP	Before	0.062 ± 0.03	0.065 ± 0.05	0.87
		Immediate	0.055 ± 0.02	0.067 ± 0.04	0.38
		30 After	0.052 ± 0.01	0.079 ± 0.02	0.24
		*P- value	0.36	0.30	
*#Significant at p<0.05*					
*Repeated measure ANOVA for **within group differences** was used.					
**Independent T-test for **between group differences** was used.					

**Fig. 2:**
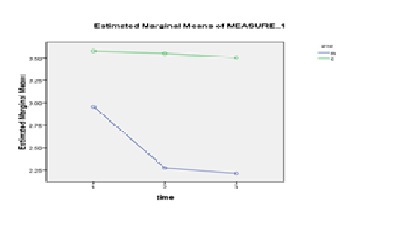
The comparison of the mean COP displacement before, immediately and 30 minutes after the application of TENS with eyes closed in both of groups

**Fig. 3:**
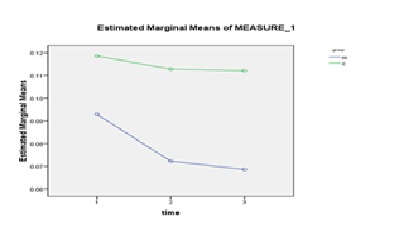
The comparison of the mean COP velocity before, immediately and 30 minutes after the application of TENS with eyes closed in both of groups

The repeated measurement for between group differences was done and the results of between groups test indicated that the variable group at COP displacement in ML direction with eyes closed (f = 4.39 , p = 0.04) and at COP velocity in ML direction with eyes closed (f = 4.8, p = 0.03) was significant (**Fig. 2**,**3**).

The within subject test indicated that there was a significant time effect, in other words, the groups changed in COP displacement and velocity over time (f = 3.31, p = 0.04, f = 4.04, p = 0.02). Moreover, the interaction of time and group was not significant (**Table 3**).

**Table 3:** The comparison of the mean COP displacement and velocity between intervention and control group before, immediately, and 30 minutes after the application of TENS with eyes closed and open

variables			TENS	Placebo-TENS	Pvalue#		
COP displacement (mm) Eye Open	ML	Before	2.92 ± 1.07	3.56 ± 1.97	0.28	0.61	0.07
		Immediate	2.40 ± 0.78	3.42 ± 1.66			
		30 After	2.32 ± 0.79	3.37 ± 2.14			
	AP	Before	2.19 ± 0.94	2.31 ± 1.27	0.61	0.16	0.42
		Immediate	2.18 ± 1.15	1.98 ± 0.79			
		30 After	1.85 ± 1.73	2.89 ± 2.27			
COP displacement (mm) Eye Closed	ML	Before	2.95 ± 0.83	3.57 ± 1.63	0.04*	0.09	0.04*
		Immediate	2.27 ± 0.45	3.54 ± 1.91			
		30 After	2.21 ± 0.52	3.50 ± 2.21			
	AP	Before	1.98 ± 1.2	2.02 ± 1.6	0.8	0.10	0.4
		Immediate	1.81 ± 0.72	2.18 ± 1.43			
		30 After	1.66 ± 0.58	2.52 ± 0.67			
COP Velocity (mm/ s) Eye Open	ML	Before	0.09 ± 0.03	0.11 ± 0.06	0.09	0.30	0.24
		Immediate	0.07 ± 0.02	0.10 ± 0.05			
		30 After	0.07 ± 0.02	0.10 ± 0.07			
	AP	Before	0.06 ± 0.03	0.07 ± 0.04	0.73	0.39	0.79
		Immediate	0.06 ± 0.03	0.06 ± 0.02			
		30 After	0.058 ± 0.05	0.07 ± 0.04			
COP Velocity (mm/ s) Eye Closed	ML	Before	0.09 ± 0.02	0.11 ± 0.05	0.02*	0.26	0.03*
		Immediate	0.07 ± 0.01	0.11 ± 0.06			
		30 After	0.06 ± 0.01	0.11 ± 0.07			
	AP	Before	0.06 ± 0.03	0.06 ± 0.05	0.75	0.14	0.41
		Immediate	0.05 ± 0.02	0.06 ± 0.04			
		30 After	0.05 ± 0.01	0.07 ± 0.02			
*#Significant at p<0.05*					
*# repeated measurement for between group differences over time was done*					

## Discussion

Our study was designed to investigate whether TENS is effective on the postural control. Displacement and velocity were used as a criterion for the estimation of postural control. The results of this study showed that low frequency TENS stimulation effectively improved postural control in LBP patients and this effect was still significant 30 minutes after the protocol.

According to our results, the application of sensory electrical stimulation in CLBP patients revealed a statistically significant improvement in postural control in mediolateral direction immediately following the application of TENS and 30minutes after it with eyes closed as compared to the baseline. It means that application of TENS decreased the displacement and velocity of COP.

This finding was consistent with the results of Laufer and Dickstein [**27**]. They measured postural control parameters during double stance with force platform. The results showed that the application of TENS induced a significant reduction in mean velocity in the mediolateral direction of the center of pressure. These findings indicated that the electrical stimulation applied to the knees might be effective in improving postural control [**27**].

In another study performed in 2006, Priplata et al. showed that COP displacement among the patients with diabetic neuropathy, stroke, and healthy elderly subjects decreased by the application of noise. Therefore, they showed that the application of subsensory mechanical noise to the feet of patients with diabetic neuropathy and stroke reduced the postural control [**28**].

The results of the present study confirmed that the displacement and velocity of COP changed with the application of electrical stimulation as compared to the use of placebo-TENS. The result showed that immediately and 30 minutes after the application of sensory electrical stimulation, COP displacement and velocity in ML direction with eyes closed significantly decreased in the intervention group compared to the controls.

Dickstein [**18**] also confirmed these results. He investigated the effect of TENS applied to the posterior aspect of the legs, on postural control during stance. The results indicated that the application of TENS decreased the postural control as expressed by a decrease in the mean of COP velocity in both the mediolateral and anterior–posterior direction. Thus, it showed that the application of low-amplitude TENS to the lower limbs decreases the postural sway during stance [**18**].

Moreover, the differences were significant only in the ML direction.It could be attributed to the impairment in controlling the anteroposterior direction due to reduced motion of the lumbar spine and increased activity of lumbopelvic muscles [**30**]. Therefore, these patients may use the muscles, which act in the frontal plane and increased activation of these muscles may induce muscle fatigue. The influence of muscle fatigue due to alteration in the trunk position and pain may induce further changing in the mediolateral direction. Luana Manreporteda higher variability in the mediolateral direction and confirmed the deficit in the anteroposterior direction in LBP patients [**31**].

In addition, hip muscles have an important role in shifting forces from the lower limb up to the spine during the upright tasks and may influence the development of LBP. A poor endurance and delay in firing of the hip abductor (gluteus medius) and hip extensor (gluteus maximus) muscles have been reported in patients with LBP [**32**]. In 2002, Nadler reported that female athletes with weakness in the left abductors were significantly more likely to develop LBP [**33**]. Therefore, the probable Gluteus Medius muscle weakness may theoretically help in developing LBP occurrence and more changes in the mediolateral direction.

Also, Janda proposed that LBP patients have a slower activation of the gluteus medius and maximus and the abdominal muscles[**34**].

The impaired balance control with eyes closed is consistent with the well-documented phenomenon of improved human balance control with visual input [**35**]. It has been demonstrated that the visual inputs play a dominant role in stance regulation [**36**]. Thus, the visual loss or visual deficit in human beings can induce different changes in the postural control.LBP is known todecreaseproprioceptive capacity [**37**,**38**], which may induce dependence on the visual system [**38**]. In 2010, Luana Man showed that LBP patients deprived fromvisual information presented an increased postural instability [**31**]. Also, the result showed no significant differences in the COP parameters with eyes open in these studies.This may be due to the fact that patients had intact information systems (visual, vestibular and somatosensory).

No studies to date have evaluated the effect of TENS on postural control in patients with CLBP but some studies showed the efficacy of TENS on the reduction of pain, disability and increased range of motion of the lumbar spine in patients with LBP following application of TENS [**39**-**42**].

Exactly how sensory electrical stimulation acts on postural control is not yet clear, but the balance improvements shown by the application of TENS are stated to be the result of an increased proprioception input. Electrical nerve stimulation improves corticomotoneural excitability by activating group Ia large muscle afferents, Ib afferents from Golgi organs, group II afferents from slow and rapidly skin afferents, and cutaneous afferent fibers [**23**].

Birmingham et al. also noted that the patients with poorer proprioceptive ability showed a greater improvement after the application of an external device [**43**]. Also, in 2002, Peurala et al. assessed the effects of electrical stimulation by using glove or sock electrodes in chronic stroke patients. They showed that sensory stimulation might enhance limb function after stroke [**44**].

Proprioceptive input from the muscles of the legs and trunk plays an important role in maintaining postural stability [**45**], suggesting that balance dysfunction in CLBP may be due to the altered proprioception feedback from the lumbar spine [**38**]. A somatosensory feedback has a necessary role in the proprioceptive system [**13**]. Therefore, the improvement in this sense following the use of TENS increases the sensory afferents.

In this study, we applied the low frequency pattern because previous studies showed beneficial effects of low frequency (1.7 and 5 Hz) and burst-type TENS for the rehabilitation of the motor impairments in patients with stroke [**46**].

There were several limitations in the current study. Firstly, the duration of the follow-up was limited. Secondly, we did not assess electromyography muscular activity of erector spine muscles and thirdly the proprioception sense of the low back spine was not measured in this study. Therefore, further research is required to assess the effect of TENS in LBP on postural control and other outcomes (proprioception) for longer time periods. 

## Conclusion

Our study was done to determine if TENS might improve postural control. There were significant differences in the displacement and velocity before, immediately and 30 minutes after the treatment with eyes closed condition. Low frequency TENS with contraction level amplitude seems to have positive effects on postural control in chronic LBP patients. Therefore, this study showed the efficacy of low frequency TENS on the improvement of postural control in patients with chronic LBP.

**Acknowledgements**

The authors would like to thank Dr. Nasrin Shokrpour for editorial assistance and Mrs. Sareh Roosta for statistical analysis at Center for Development of Clinical Research of Nemazee Hospital. This article is extracted from a Master of Sciences thesis Tahere Rezaeian, MSc, proposal number 6511.
